# The relationship between human adenovirus 36 and obesity in Chinese Han population

**DOI:** 10.1042/BSR20180553

**Published:** 2018-07-06

**Authors:** Yan Zhou, Qi Pan, Xiaoxia Wang, Lina Zhang, Fei Xiao, Lixin Guo

**Affiliations:** 1 Beijing Hospital, National Center of Gerontology, Beijing 100730, China; 2 Key Laboratory of Geriatrics, Department of Pathology, Beijing Hospital, National Center of Gerontology, Beijing 100730, China

**Keywords:** BMI, human adenovirus 36, obesity, seropositivity

## Abstract

The study aimed to explore the prevalence of human adenovirus-36 (HAdV-36) infection and the association of HAdV-36 with obesity in Chinese Han population. A qualitative determination using ELISA was performed to determine by duplication of the antibodies to HAdV-36 in the serum samples. Logistic regression analysis was used to analyze the association between HAdV-36 seropositivity and obesity. The overall HAdV-36 seroprevalence was 49.8% amongst 824 participants. The prevalence of HAdV-36 seropositive was 42.9 and 51.4% in the obese and non-obese participants, respectively, which was not statistically significant (*P*=0.05). There were significant differences in the anthropometric and biochemical parameters observed between the two groups except for height (*P*=0.067) and total cholesterol (TC) (*P*<0.29). After the adjustment for age and gender, HAdV-36 seropositivity was a protective factor for obesity (odds ratio (OR) = 0.69, 95% confidence intervals (95% CI) = 0.48–0.97, *P*=0.03). In the male population, the adjusted OR for AD-36 antibody-positive status was statistically decreased for obese adults (OR = 0.59; 95% CI = 0.39–0.91; *P*=0.02). However, the similar result was not obtained in the female population (OR = 0.90; 95% CI = 0.48–1.67; *P*=0.73). We found a high prevalence of HAdV-36 infection in China and significant association between HAdV-36 infection and obesity or weight gain after the adjustment for age and gender. The HAdV-36 infection may be related to the weight loss in Chinese Han population, especially in the male group, which needs to be further confirmed.

## Introduction

Environmental and genetic influences have been identified as the main factors for obesity [1,2]. As obesity is regarded as a primary pathogenic factor for many acute and chronic diseases, including hypertension, coronary heart disease (CHD), stroke, and some types of cancer, which produce high, severe burden of diseases on the patients and their relatives, therefore, prevention of obesity becomes an important public health issue throughout the world.

Previously, it was reported that the viral infection may be related with the obesity through the animal models [3], while human adenovirus 36 (HAdV-36) has been shown to cause obesity in multiple animal species [4,5] . It has been reported that HAdV-36 infection in animals resulted in increasing proliferation and differentiation of preadipocytes and lipid accumulation in mature adipocytes [5–7].

The prevalence of HAdV-36 infection in humans and the association with obesity/metabolic abnormalities were also reported in different countries [8–13]. Approximately 30% of obese and 11% of non-obese humans had neutralizing antibodies to HAdV-36 which were associated with reductions in serum cholesterol and TG [14,15]. In Italy, HAdV-36 infection was more common in obese and the prevalence ranged from 29 to 65% [16,17]. In Swedish, HAdV-36 infection was linked with children obesity, severe obesity in female adults, and lower risk of high blood lipid levels [12]. In Children, the prevalence of antibodies to HAdV-36 was higher in obese than in non-obese in U.S.A. and South Korea [18,19].

In previous studies, it was reported that obesity may be induced after infecting with HAdV-36. [13,20–23], but in Chinese Han population there was no related study about this relationship. The current study aimed to explore the prevalence of HAdV-36 infection and the association of HAdV-36 with obesity in Chinese Han population.

## Materials and methods

### Participants

All methods were performed in accordance with the relevant guidelines and regulations. The data of the study population were collected from January 2015 to May 2016 in Beijing hospital for physical examination of adults. The study excluded those who were less than 18 years old and more than 80 years old, non-Han population. Participants with the following characteristics were also excluded: (i) using medication that causes weight loss or weight gain, (ii) genetic conditions associated with being overweight or obesity, and (iii) illness that may affect weight. Clinical samples, epidemiological information, and the clinical data were collected. Informed written consent was obtained from all participants before enrollment in the study. Approval for the study was obtained from the Research Ethics Committee of Beijing Hospital. For the sample size calculation, reference data [24] showed a 47% AD-36 antibody positive rate in the obese group and 32.5% in the non-obese group. Set at significance level of 0.05, the power of 80%, *n*=356 patients (*n*=178 patients in each study group) were needed.

### Clinical and anthropometric measurements

Body weight and height were determined after the removal of shoes and outdoor clothing. Body weight was measured to the nearest 0.10 kg by electronic scale using a Tanita Body Composition Monitor (Tanita BC-553, Arlington, VA). Body height was measured to the nearest 0.10 cm using Wall Mounted Stadiometer (Novel Products Inc, Rockton, IL, U.S.A.). Body mass index (BMI) was calculated by prepregnancy weight (kg) divided by the square of height (m). The participants were categorized into obese (BMI: over 28) and non-obese (BMI: less than 28) groups according to the criteria of weight for adults in the Health industry standard of China (WS/T 428-2013). The body circumferences were measured using a diameter tape accurate to within ±0.10 cm (Seca 201, Hamburg, Germany). The duplicate measures were averaged. The blood pressure was measured twice in a row at the right arm of participants at 1-min intervals with an aneroid sphygmomanometer (Riester CE 0124, Jungingen, Germany).

### Laboratory measurements

Venous blood samples and urine were collected after overnight fasting. Biochemical parameters, such as LDL-cholesterol (LDL-c), total cholesterol (TC), HDL-cholesterol (HDL-c), triglycerides (TG), fasting glucose levels, GPT, GOT, total bilirubin (TBIL), direct bilirubin, ALP, glutamyltranspeptidase creatinine, uric acid, and carbamide were analyzed immediately using a semiautomated apparatus (Cobas Mira). Insulin levels were measured using a commercially available ELISA (GenWay INS-ELISA kit). The HOMA index to determine insulin resistance was calculated using the formula: (fasting insulin (U/ml) × fasting glucose (mmol/l))/22.5.

A qualitative determination using ELISA was used to determine by duplicate the antibodies to HAdV-36 in the serum samples (Human ADV-36 Ab ELISA Kit, TSZ Biosciences, Balfour St, Lexington, MA, U.S.A.).

### Statistical analysis

For continuous variables that follow normal distribution, the Student’s *t* test was used to analyze the difference between the two groups while for other variables, non-parametric tests Kruskal–Wallis test and Chi-square test were used in the research. Binary logistic regression was used to analyze the association between HAdV-36 seropositivity and obesity. For the non-normal distributed covariates, instead of the actual value, the quintile value was used to make the model more robust. Backwards elimination variable selection method was used to select significant factors. Odds ratios (ORs) and their 95% confidence intervals (CIs) were estimated using maximum likelihood methods. Two-sided *P*-value less than 0.05 was considered statistically significant. Data analysis was performed using SAS software 9.1.3 (SAS Institute, Cary, NC, U.S.A.).

## Results

Total 824 participants (486 male and 338 female) were included. The median age of participants was 46.0 years, with IQR of (37.0, 55.0) years and the median BMI was 24.6 kg/m^2^, with IQR of (22.5, 27.2) kg/m^2^. The overall HAdV-36 seroprevalence was 49.8%. A comparison between non-obese and obese groups was shown in Table 1. The AD-36 seropositive rate of obese group was 42.86%, which was lower than the non-obese group (51.43%), but the difference was not statistically significant (*P*=0.05). There were significant differences in anthropometric and biochemical parameters observed between groups except for height (*P*=0.07) and TC (*P*=0.29).

**Table 1:** Characteristics of the study population

	Non-obese	Obese	*P*
Age (years)	47.02 ± 12.92	47.54 ± 12.87	0.645
Sex			0.001
Male	373 (56.26)	113 (70.19)	
Female	290 (43.74)	48 (29.81)	
Height (kg)	166.69 ± 7.87	167.98 ± 8.70	0.068
Weight (cm)	65.89 ± 9.98	85.12 ± 9.62	0.000
BMI (kg/m^2^)	23.62 ± 2.48	30.11 ± 1.90	0.000
WC (cm)	82.91 ± 8.52	97.26 ± 7.50	0.000
Systolic BP (mmHg)	124.95 ± 18.80	133.59 ± 16.50	0.000
Diastolic BP (mmHg)	77.40 ± 9.01	83.60 ± 10.71	0.000
TG (mg/dl)	1.53 ± 1.07	1.95 ± 1.20	0.000
TC (mg/dl)	4.85 ± 0.90	4.94 ± 0.80	0.292
LDL-c (mg/dl)	3.01 ± 0.74	3.18 ± 0.65	0.000
HD-c (mg/dl)	1.29 ± 0.30	1.13 ± 0.23	0.000
HAdV-36			
Positive	341 (51.43)	69 (42.86)	0.051
Negative	322 (48.57)	92 (57.14)	
GPT (U/l)	21.29 ± 13.58	30.47 ± 18.60	0.000
GOT (U/l)	26.65 ± 7.19	29.99 ± 9.20	0.000
TBIL (mmol/l)	13.52 ± 5.11	13.21 ± 5.17	0.482
Direct bilirubin (mmol/l)	4.40 ± 1.72	4.39 ± 1.78	0.977
Uric acid (μmol/l)	309.05 ± 84.71	363.89 ± 82.05	0.000
Glucose (mmol/l)	5.32 ± 0.88	5.47 ± 0.83	0.048
Carbamide	116.63 ± 144.23	169.22 ± 176.74	0.000

WC, waist circumference; BP, blood pressure.

According to the HAdV-36 antibody status, the characteristics of participants are shown in Tables 2.1 and 2.2 by obese group. For all the participants and the non-obese group alone, the HAdV-36 antibody-positive populations were younger, higher, lower in diastolic BP and carbamide, and higher in GOT. The HAdV-36 antibody-positive population in non-obese group is also higher in uric acid, which was not shown in obese group and the two groups combined. In the obese group, the characteristic was not much different regardless of the HAdV-36 status except one interesting finding that adults with HAdV-36 antibody-positive in obese adults have a finer waist circumference compared with those with HAdV-36 antibody-negative (Table 2.1, *P*=0.03). In obese group, non-obese group, and two groups combined, there was no statistically significant difference in BMI, TG, TC, LDL, and HDL between HAdV-36 antibody-positive and HAdV-36 antibody-negative groups (all *P*>0.05).

**Table 2.1:** The characteristics of non-obese and obese adults according to the HAdV-36 antibody status*

	Non-obese			Obese		
	HAdV-36^−^	HAdV-36^+^	*P*	HAdV-36^−^	HAdV-36^+^	*P*
*n*	322	341	—	92	69	—
Age (years)	47.0 (39.0, 57.0)	45.0 (36.0, 53.0)	0.04	46.0 (38.0, 56.5)	47.0 (37.0, 57.0)	0.79
Male	168 (52.2%)	205 (60.1%)	0.04	65 (69.2%)	48 (69.6%)	0.96
Height (cm)	166.0 (159.4, 172)	167.6 (162.3, 172.9)	0.01	170.3 (161.8, 173.9)	168.3 (163.3, 173.0)	0.78
Weight (kg)	64.2 (57.4, 72.0)	67.1 (60.4, 74.2)	0.01	86.7 (78.1, 91.2)	83.9 (78.6, 90.8)	0.57
BMI (kg/m^2^)	23.7 (21.8, 25.4)	23.8 (22.2, 25.6)	0.25	29.9 (28.8, 31.0)	29.5 (28.4, 30.6)	0.12
WC (cm)	83.0 (77.0, 89.0)	84.0 (77.0, 89.0)	0.61	98.5 (93.5, 104.0)	96.0 (90.0, 101.0)	0.03
Systolic BP (mmHg)	124.0 (113.0, 135.0)	122.0 (111.0, 133.0)	0.07	132.0 (123.0, 146.5)	131.0 (122.0, 143.0)	0.46
Diastolic BP (mmHg)	77.0 (72.0, 83.0)	76.0 (72.0, 83.0)	0.05	82.0 (77.0, 89.0)	83.0 (78.0, 88.0)	0.87
TG (mg/dl)	1.2 (0.8, 1.8)	1.2 (0.9, 2.0)	0.07	1.6 (1.2, 2.2)	2.0 (1.1, 2.6)	0.45
TC (mg/dl)	4.8 (4.2, 5.4)	4.8 (4.3, 5.3)	0.51	4.9 (4.3, 5.3)	5.1 (4.5, 5.5)	0.07
LDL-c (mg/dl)	2.9 (2.5, 3.4)	3.0 (2.6, 3.4)	0.29	3.2 (2.6, 3.6)	3.4 (2.9, 3.7)	0.08
HDL-c (mg/dl)	1.3 (1.1, 1.5)	1.2 (1.1, 1.4)	0.43	1.1 (0.9, 1.3)	1.1 (1.0, 1.3)	0.55
GPT (U/l)	18.0 (13.0, 24.0)	18.0 (14.0, 25.0)	0.73	26.5 (18.5, 37.0)	24.0 (19.0, 32.0)	0.41
GOT (U/l)	25.0 (21.0, 29.0)	26.0 (22.0, 30.0)	0.01	28.0 (24.5, 32.0)	28.0 (24.0, 35.0)	0.84
TBIL (mmol/l)	12.6 (9.6, 16.4)	13.1 (9.8, 16.1)	0.78	12.2 (9.8, 16.0)	12.1 (9.7, 14.6)	0.76
Direct bilirubin (mmol/l)	4.0 (3.1, 5.4)	4.1 (3.3, 5.4)	0.85	4.3 (3.1, 5.3)	4.0 (3.3, 4.9)	0.53
Uric acid (μmol/l)	289.5 (238.0, 366.0)	313.0 (264.0, 369.0)	0.01	361.0 (299.5, 419.0)	375.0 (299.0, 426.0)	0.99
Glucose (mmol/l)	5.2 (4.9, 5.5)	5.2 (4.9, 5.6)	0.35	5.4 (5, 5.9)	5.3 (5.0, 5.6)	0.16
Carbamide	191.5 (4.9, 268.0)	5.2 (4.0, 188.0)	<0.01	77.0 (4.7, 314.0)	8.1 (4.8, 319.0)	0.97

* Variables are presented as median (IQR).

**Table 2.2:** The characteristics of all adults according to the HAdV-36 antibody status*

	HAdV-36^−^	HAdV-36^+^	*P*
*n*	414	410	—
Age (years)	47.0 (39.0, 57.0)	45.0 (36.0, 54.0)	0.04
Male	233 (56.3%)	253 (61.7%)	0.11
Height (cm)	166.4 (159.8, 172.7)	167.8 (162.3, 173.0)	0.04
Weight (kg)	67.5 (60.2, 78.7)	69 (61.5, 77.5)	0.16
BMI (kg/m^2^)	24.7 (22.4, 27.7)	24.6 (22.5, 26.9)	0.53
WC (cm)	87.0 (79.0, 93.0)	85.0 (79.0, 91.0)	0.20
Systolic BP (mmHg)	126.0 (115.0, 138.0)	123.5 (113.0, 134.0)	0.02
Diastolic BP (mmHg)	78.0 (73.0, 85.0)	78.0 (71.0, 84.0)	0.03
TG (mg/dl)	1.3 (0.9, 1.9)	1.3 (0.9, 2.1)	0.18
TC (mg/dl)	4.8 (4.3, 5.4)	4.9 (4.3, 5.4)	0.94
LDL-c (mg/dl)	3.0 (2.5, 3.5)	3.0 (2.6, 3.5)	0.18
HDL-c (mg/dl)	1.2 (1.1, 1.4)	1.2 (1.1, 1.4)	0.99
GPT (U/l)	19.0 (14.0, 27.0)	19.0 (14.0, 26.0)	0.59
GOT (U/l)	25.0 (22.0, 30.0)	26.0 (22.0, 31.0)	0.04
TBIL (mmol/l)	12.6 (9.7, 16.2)	12.8 (9.8, 15.8)	0.88
Direct bilirubin (mmol/l)	4.1 (3.1, 5.4)	4.1 (3.3, 5.3)	0.95
Uric acid (μmol/l)	307.0 (250.0, 381.0)	318.0 (268.0, 385.0)	0.06
Glucose (mmol/l)	5.2 (4.9, 5.6)	5.2 (4.9, 5.6)	0.94
Carbamide	186.0 (4.8, 289.0)	5.3 (4.2, 248.0)	<0.01

* Variables are presented as median (IQR).

To determine how HAdV-36 affects obesity, the binary logistic regression was performed where the variable obesity was the dependent variable. For HAdV-36 antibody status, the unadjusted OR was not statistically significant between non-obese and obese adults (*P*=0.052). After the adjustment for age and gender, HAdV-36 seropositivity was a protective factor for obesity (OR = 0.69; 95% CI = 0.48–0.97; *P*=0.03). To further examine relationship, we added the significant characteristics in Tables 2.1 and 2.2 into the regression model. After backwards variable selection, only diastolic BP and uric acid were left in the model, HAdV-36 seropositivity is still a protective factor but only significant at significance level 0.1. The results are shown in Table 3.

**Table 3:** OR and 95% CI for non-obese and obese adults according to the HAdV-36 antibody status

	OR	95% CI	*P*
HAdV-36^−^	Reference		
HAdV-36^+^ (unadjusted)	0.71	0.50–1.00	0.05
Adjusted for age and sex	0.69	0.48–0.97	0.03
Adjusted for diastolic BP and uric acid	0.70	0.48–1.02	0.06

Considering the imbalance between the proportion of male and female in non-obese and obese groups, we performed binary logistic regression analysis for different sexes. In the male population, there was statistically significant difference between the obese and non-obese adults without any adjustments (OR = 0.61; 95% CI = 0.40–0.93; *P*=0.02). After adjusting for age, the difference between two groups in male was still statistically significant (OR = 0.58; 95% CI = 0.39–0.91; *P*=0.02). After the adjustement for diastolic BP and uric acid, HAdV-36 seropositivity is still a protective factor for male but only significant at significance level of 0.1. However, unadjusted and adjusted OR for HAdV-36 antibody status was not statistically significant between non-obese and obese female adults (*P*>0.05) shown in Table 4.

**Table 4:** OR and 95% CI for non-obese and obese adults in different sexes according to the HAdV-36 antibody status

	Male			Female		
	Non-obese	Obese	*P*	Normal	Obese	*P*
	OR	95% CI		OR	95% CI	
HAdV-36^−^	Reference			Reference		
HAdV-36^+^ (unadjusted)	0.61	0.40–0.93	0.02	0.88	0.48–1.63	0.69
Adjusted for age	0.59	0.39–0.91	0.02	0.90	0.48–1.67	0.73
Adjusted for diastolic BP and uric acid	0.66	0.42–1.04	0.07	0.78	0.39–1.55	0.47

HAdV-36 seropositivity was analyzed with different number of metabolic alterations (Table 5). The percentages of metabolic abnormalities with over 3 were 24.6 and 27.6% in the HAdV-36 seropositive and seronegative groups, respectively. No significant differences were found in the numbers of metabolic abnormalities between the two groups (all *P*>0.05).

**Table 5:** HAdV-36 seropositivity and number of metabolic abnormalities

Characteristics	AD-36^−^	AD-36^+^	OR (95% CI)	*P*-value
0	19 (4.6)	16 (3.9)	1.000	
1	162 (39.1)	164 (40.0)	1.26 (0.62, 2.54)	0.52
2	119 (28.7)	128 (31.2)	1.37 (0.67, 2.79)	0.39
3	88 (21.3)	67 (16.3)	0.98 (0.47, 2.07)	0.97
4	23 (5.6)	32 (7.8)	1.72 (0.73, 4.07)	0.21
5	3 (0.7)	2 (0.5)	0.82 (0.12, 5.54)	0.84

OR, odds ratio.

## Discussion

Some meta-analysis reported that HAdV-36 infection was associated with obesity [22,23,25]. Observed from those references, the HAdV-36 infection may contribute to the obesity development or the weight gain. However, we did not obtain the similar conclusion in Chinese Han population. In contrast, the result obtained in our research showed that the HAdV-36 infection may not cause the weight gain, which may be a protective factor for male adult obesity in Chinese Han population.

Few studies focussed on the effect of HAdV-36 infection on obesity in China. This is the first to report the prevalence of HAdV-36 infection and explore the association with obesity in Chinese Han population. There was only one research in this area in Chinese Uygur population [24]. The results showed the prevalence of HAdV-36 infection was 42.9% in the obese group, which was similar to those in Chinese Uygur population (47.0%). However, there was a big difference in the prevalence of HAdV-36 infection between the Chinese Han and Uygur non-obese population (51.4 compared with 32.5%). In other countries, most had lower prevalence rates, such as 30% in obese and 11% in non-obese in U.S.A. [15], 29 and 14% in Korea [26], and 5.5% in Dutch and Belgian individuals [11]. In Italy, Trovato et al. [16,17] noted that a prevalence of HAdV-36 antibodies was more than 40%. A potential explanation of the differences may be the problems with the assays [15]. In our opinion, the population and regional differences may also contribute to this. The distributions of HAdV types differ from time and place, for example HAdV-14 and HAdV-55 occurred in China only in the recent 5 years [27,28]. Maybe the HAdV-36 infection had this characteristic in the world and the susceptibility was also different in diverse races.

In our study, we did not find a positive relationship between HAdV-36 infection and obesity risk but a negative one. The OR was lower than those in other studies which also did not demonstrate the causative and correlative role of HAdV-36 infection and obesity [10,29,30]. Of course, this result was not consistent to most of the studies [8,9,19,20,30–33]. Owing to the difference of region, age, and assay method, the results in different studies were not comparable. Especially, inadequate sample size and unrepresentative subjects may result in an untrusted conclusion [23]. The high prevalence of HAdV-36 infection in China indicated the high susceptibility to HAdV-36 in Chinese population, which may confuse the association with obesity. Except for considering the difference of selected population, the conclusion in the study needs to be confirmed by the cross-sectional or cohort studies with a larger sample size. We did not find the significant association of HAdV-36 with weight gain, which was consistent to the new meta-analysis result [23]. In one study, HAdV-36 positive group showed only a slightly lower response to weight loss than the HAdV-36 negative group, with the only statistically significant difference being a smaller reduction in BMI percentile [34]. It’s difficult to say that the change of BMI was caused by the HAdV-36 infection. Maybe a long follow-up study could resolve this question.

There were some limitations in the present study. The ELISA method was not the gold standard for the diagnosis of previous or current HAdV-36 infection, which may result in a bias in the detection of HAdV-36. All the participants came from the same hospital, so the representativeness of samples may have a bias on the current result. Only adults (age: >18) were recruited and the children (age: ≤18) were excluded, so that we cannot do some research on the prevalence of HAdV-36 infection amongst children. We did not consider the potential factors influencing obesity or weight loss, which may also affect the results.

In conclusion, the results obtained in our research showed that there was a higher prevalence of HAdV-36 infection in China than in other countries and significant association between HAdV-36 infection and obesity or weight gain in Chinese Han population without considering the gender difference. In other words, the HAdV-36 infection may be related to the weight loss in Chinese Han population, especially in the male group, which needs to be further confirmed.

## Abbreviations

ALP: alkaline phosphatase
BMI: body mass index
95% CI: 95% confidence interval
GOT: glutamic oxalo acetic transaminase
GPT: glutamic-pyruvic transaminase
HAdv-36: human adenovirus-36
HDL-c: HDL-cholesterol
LDL-c: LDL-cholesterol
OR: odds ratio
TBIL: total bilirubin
TC: total cholesterol
TG: triglyceride
